# Regional differences in lumbar spinal posture and the influence of low back pain

**DOI:** 10.1186/1471-2474-9-152

**Published:** 2008-11-18

**Authors:** Tim Mitchell, Peter B O'Sullivan, Angus F Burnett, Leon Straker, Anne Smith

**Affiliations:** 1 School of Physiotherapy, Curtin University of Technology, Kent St, Bentley, Western Australia, Australia; 2 School of Exercise, Biomedical and Health Sciences, Edith Cowan University, Joondalup Drive, Joondalup, Western Australia, Australia

## Abstract

**Background:**

Spinal posture is commonly a focus in the assessment and clinical management of low back pain (LBP) patients. However, the link between spinal posture and LBP is not fully understood. Recent evidence suggests that considering regional, rather than total lumbar spine posture is important. The purpose of this study was to determine; if there are regional differences in habitual lumbar spine posture and movement, and if these findings are influenced by LBP.

**Methods:**

One hundred and seventy female undergraduate nursing students, with and without LBP, participated in this cross-sectional study. Lower lumbar (LLx), Upper lumbar (ULx) and total lumbar (TLx) spine angles were measured using an electromagnetic tracking system in static postures and across a range of functional tasks.

**Results:**

Regional differences in lumbar posture and movement were found. Mean LLx posture did not correlate with ULx posture in sitting (r = 0.036, p = 0.638), but showed a moderate inverse correlation with ULx posture in usual standing (r = -0.505, p < 0.001). Regional differences in range of motion from reference postures in sitting and standing were evident. BMI accounted for regional differences found in all sitting and some standing measures. LBP was not associated with differences in regional lumbar spine angles or range of motion, with the exception of maximal backward bending range of motion (F = 5.18, p = 0.007).

**Conclusion:**

This study supports the concept of regional differences within the lumbar spine during common postures and movements. Global lumbar spine kinematics do not reflect regional lumbar spine kinematics, which has implications for interpretation of measures of spinal posture, motion and loading. BMI influenced regional lumbar posture and movement, possibly representing adaptation due to load.

## Background

Low back pain (LBP) remains one of the most expensive medical conditions in manual workers including nurses [1]. Opinion remains divided regarding optimal LBP management [2]. Although retraining "ideal" spinal posture is a common component of the clinical management of non-specific LBP patients [3,4], the direct relationship between spinal posture and LBP still remains unclear.

Evidence of both a relationship [5,6], or no relationship [7,8] between posture and LBP has been reported in previous *in-vivo *posture studies. These conflicting findings may be due to posture being relevant to LBP in some populations but not others, or alternatively may be explained by methodological differences. When investigating posture, measures need to possess sufficient discriminative validity [9]. Clinically, LBP patients report more pain in the lower lumbar (LLx) spinal segments than upper lumbar (ULx) segments [10,11]. This is consistent with a greater degree of degeneration being evident in the LLx spinal segments [12,13], which is thought to be due to the greater mechanical stress through these segments [14]. Given some individual lumbar spinal segments show greater degenerative changes than other lumbar segments, the notion of the lumbar spine as a homogenous region may not provide a true reflection of pain and function in this region.

To date, the concept of considering the motion and function of the lumbar spine in terms of LLx and ULx regions has been proposed [15], but not widely investigated [9]. The majority of studies examining LBP have not considered lumbar spinal posture in separate regions, which may help explain the consensus of no direct link between spinal posture and LBP [16,17]. Other factors including gender [8] and BMI [18], which are known to influence posture, may also confound this issue. However, there is emerging *in-vivo *evidence of links between posture and LBP. Dankaerts and colleagues showed differences in usual sitting posture between LBP patients and healthy controls [6]. Importantly, these differences were only evident when the lumbar spine was considered as separate regions (LLx and ULx), and when LBP subjects were sub-classified according to directional pain provocation patterns [6].

The concept of considering regional motion and function of the lumbar spine during functional tasks has only recently been investigated. Gill and associates identified the importance of considering the lumbar spine as having separate regions, rather than viewing it as a rigid section, when measuring spinal lifting patterns [19]. Their recent study examining healthy subjects has shown a lack of variation of LLx spine posture when commencing lifting, irrespective of both the lifting technique used, or the distance the load is away from subject's feet [19]. In this study, movement variation when lifting was found to occur in the ULx and mid thoracic spine, rather than the LLx spine. These findings in healthy controls are yet to be examined in a LBP population.

Further investigation of regional differences in ULx and LLx spine function across different functional tasks relevant to specific work populations is required, as many LBP patients report symptom aggravation across a number of activities or postures other than just lifting [20]. The primary hypothesis of this study was that regional lumbar spine differences would be evident in standing and sitting postures, as well as for spinal angles and range of motion during functional tasks.

The aims of this study were to determine:

1. whether regional (LLx/ULx) differences exist in spinal sagittal; static posture angles, range of motion and dynamic spinal angles during functional tasks.

2. if the nature of these differences are similar in subjects with and without a history of LBP.

## Methods

### Design

This cross-sectional study was part of a larger prospective study into patterns of LBP in nursing students. This current study examined the LBP characteristics and spinal kinematics across a range of static postures and functional tasks of female undergraduate nursing students.

### Sample

Data were collected on 170 female undergraduate nursing students recruited via personal invitation from two undergraduate university nursing programs. Subjects were aged between 18 and 35 years and were in their second or third year of their programs at the time of the study. Ethical approval to conduct the study was granted from Curtin University of Technology and Edith Cowan University ethics committees, and written informed consent from subjects was obtained.

### Protocol

Subjects were excluded if they had; an inability to understand written or spoken English, presence of other conditions affecting the spine or lower limbs including inflammatory disorders, neurological diseases or metastatic disease, pregnancy or less than 6 months post-partum, or inability to assume the test postures. Subjects both with and without a history of LBP were included in the study. As acute LBP has been shown to influence spinal posture [21] and motor control [22], subjects who had LBP which limited their performance of the test procedures (pain greater than 3 out of 10 on a VAS at the time of testing) were excluded (1 subject).

### LBP characteristics

Based on a previous survey of LBP in a similar nursing student sample [23], a range of LBP severity was expected. To investigate the influence of LBP, subjects were divided into three LBP categories; No LBP, Minor LBP and Significant LBP. Considering the multifactorial influences of LBP [24], and variance in prevalence based on LBP definition [25], Significant LBP group allocation was defined by a combination of indicators across a range of domains based on previous LBP research. These indicators were:

1. Lifetime LBP Severity. Subjects were asked to rate their worst ever LBP on a visual analogue pain scale (> 4/10. Based on mean episodic LBP severity data [26]).

2. Duration of LBP in previous 12 months. Taken from Nordic LBP Questionnaire [27] (>1 week. To differentiate subjects with a single very short LBP episode of high severity).

3. LBP requiring treatment or medication or a reduction in activity in the past 12 months [28].

4. LBP disability levels at the time of testing measured by the Oswestry Disability Index (ODI) [29], (>20% based on mean ODI score for primary LBP of 26% [30]).

Subjects who scored above the designated cut off score in at least three of the four categories were deemed to have Significant LBP. The remaining LBP subjects who reported some pain in the previous 12 months, but did not satisfy the criteria for Significant LBP were considered as having Minor LBP (Table 1).

**Table 1:** Subject Demographics and LBP Characteristics

	**No LBP (n = 36)**	**Minor LBP (n = 81)**	**Significant LBP (n = 53)**
**Age (mean + SD, years)**	21.7 ± 3.5	22.0 ± 4.2	23.9 ± 5.1
**BMI (mean + SD, kg/m^2)^**	21.9 ± 2.8	23.3 ± 4.3	23.1 ± 3.4
**Lifetime highest VAS (mean + SD,/10)**	0	3.9 ± 2.3	6.6 ± 1.6
**Annual LBP Duration (range, days)**	0	1–7	8–30
**Requiring treatment, medication or activity reduction past 12-months (%)**	0	44.4	96.2
**Oswestry Disability Index (mean + SD)**	0	10.4 ± 6.6	21.2 ± 9.2

BMI = body mass index. VAS = visual analogue scale.

**Figure 1:**
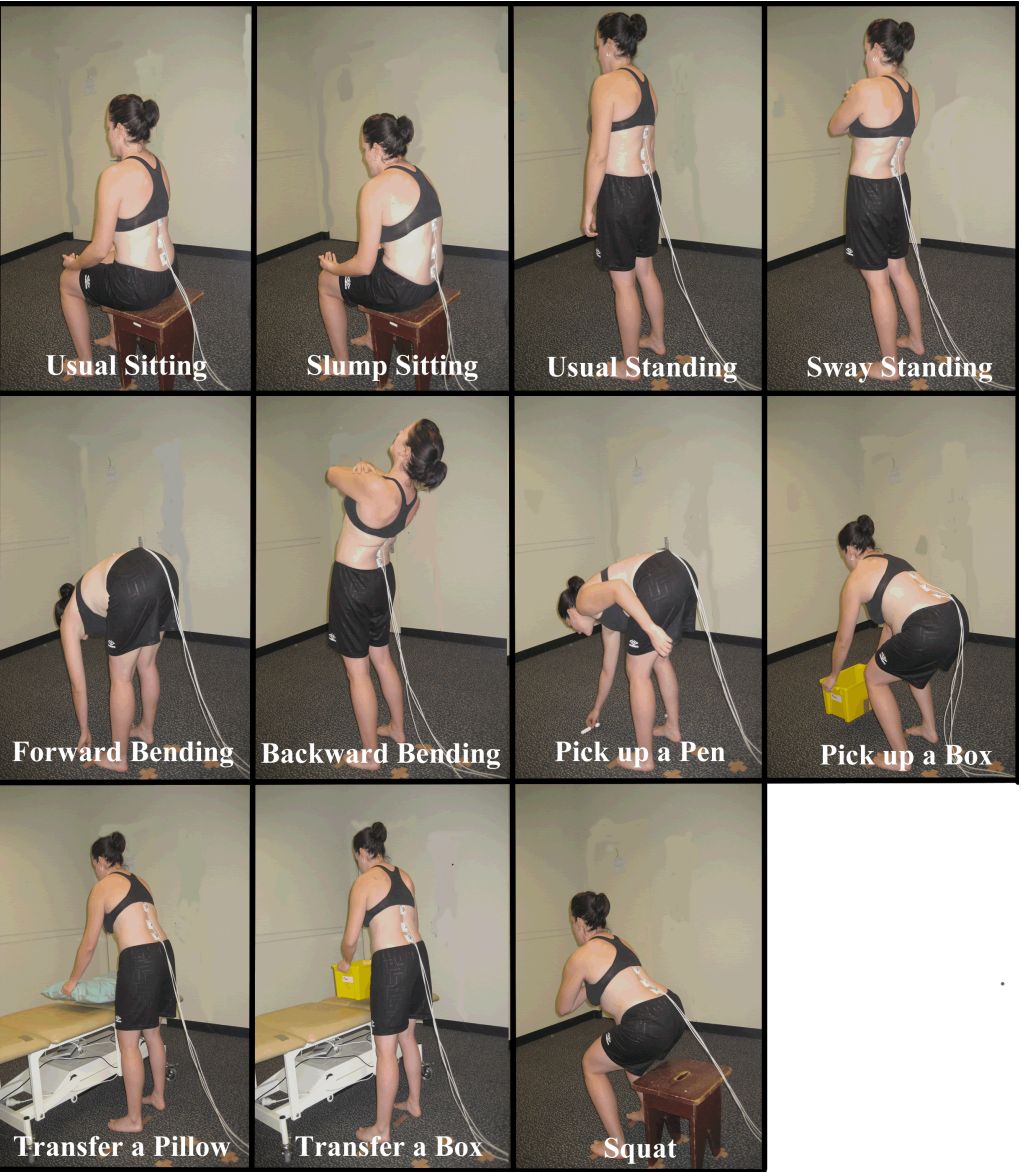
Test postures.

Subjects attended a single testing session at their university. A modified version of the Nordic Low Back Pain Questionnaire [27] was used to determine LBP history, frequency and severity. LBP disability levels were measured using the ODI. BMI was calculated from height and weight measures to control for its known influence on spinal posture and motion [18]. The static spinal postures measured were usual sitting and usual standing. Sagittal spinal range of motion were measured as the difference between usual sitting and maximal slumped sitting and usual standing and; sway standing, maximal forward bending and maximal backward bending in standing. Peak sagittal angles were measured during a range of functional tasks chosen with consideration of likely repetitive movements and sustained postures associated with university study and nursing duties. Test postures are shown in Figure 1.

### Static Sitting and Standing Posture

It is acknowledged that measuring true "usual" posture is difficult in the laboratory setting. However, subjects were covertly observed when completing questionnaires prior to physical testing to gain an idea of their "usual" sitting posture, and to ensure a similar posture was adopted during testing. Further, subjects were not aware when the "usual" standing and sitting measures were being recoded, as they performed a number of tasks that involved sitting or standing as the starting position. Usual sitting and standing postures were measured as follows using a previously described protocol [6]:

1. Subjects were asked to sit on a stool, which was selected to allow their thighs to be parallel with the floor and knees flexed at 90°. No direction of how to sit or an indication of what was being measured was provided. This position was recorded for five seconds as their usual sitting posture (defined as the sitting posture they would usually adopt during unsupported sitting).

2. Subjects were asked to stand comfortably at a predetermined position. Whilst no specific instruction of how to stand was given, all subjects stood with their feet parallel. This position was recorded for five seconds as their usual standing posture (defined as the standing posture they would usually adopt during habitual unsupported standing).

### Range of motion in sitting and standing

1. From the usual sitting position subjects were then assisted into their end of range lumbar flexion sitting posture for five seconds by an experienced therapist using standardised cues of asking the subject to "slouch" and using hand cues on the lateral shoulder and pelvis to guide posterior pelvic tilting.

2. Sway standing posture was defined as subject's relaxed standing posture with the pelvis translated anteriorly relative to the trunk. All subjects were guided into this position from their usual standing position for five seconds by the same experienced therapist. Excellent reliability of positioning subjects in sway posture has been shown previously [31].

3. Subjects were then asked to bend forwards as far as possible from standing, with their knees straight, and a five second recording in this position was defined as maximal forward bending.

4. Similarly, maximal backward bending was measured by asking subjects to then bend backwards as far as possible for five seconds, keeping their feet stationary.

All posture and range of motion measures were repeated three times.

### Functional tasks

1. While in the standing position, a pen was placed in front of subjects and they were asked to pick it up. Subjects were directed to pick up the pen as if they had just dropped their own pen on the floor and needed to retrieve it. This test was performed once.

2. Subjects were then directed to pick up a moderate (5 kg) load in a box with handles 20 cm above floor height. No cues were given regarding how to pick up the box. This and subsequent tasks were repeated three times.

3. An adjustable bed was then set at a height 10 cm above each subject's superior patella margin as a standardised height. The task involved transferring a pillow from left to right a distance of 75 cm, then back to the starting position. Subjects initially stood at the mid point between the pillow and target position marked on the bed, then were asked to transfer the load, with no specific directions regarding how to lift.

4. The task involving transferring a pillow was then repeated using a 5 kg box.

### Squatting

Subjects were seated on a stool, with thighs parallel and knees flexed at 90°, and their arms folded across their chest. Subjects were then asked to adopt a squat position with their buttocks just clear of the stool, by an experienced therapist using standardised cues. This test was also used for a measure of leg muscle endurance, so only one trial was conducted. Subject's lumbar spine posture was recorded throughout the squat test, with a five second Fastrak™ data sample taken as their squat position once their position was stable after rising from the stool.

### LLx, ULx and TLx angle measurement

**Figure 2:**
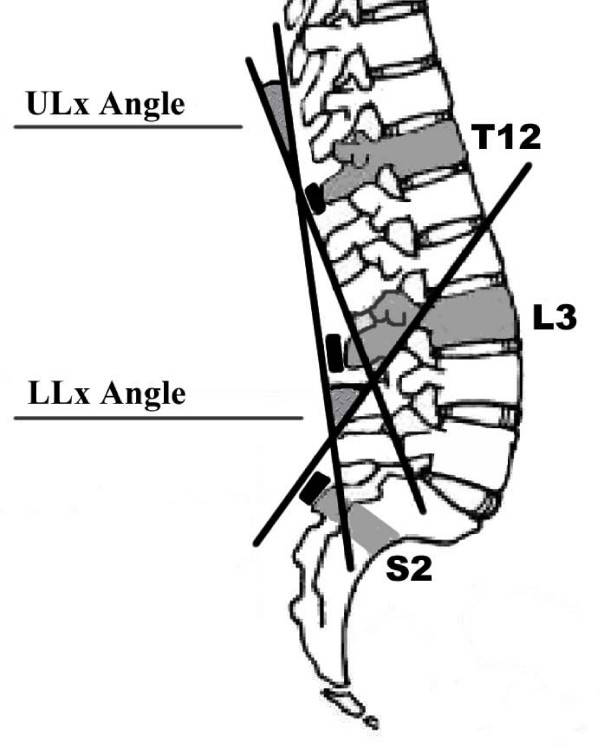
**Spinal model used for the calculation of lumbar angles**. LLx = lower lumbar; ULx = upper lumbar. Total lumbar angle is the angle formed between the tangents from the sensors at T12 and S2.

Lumbar spine sagittal plane (flexion/extension) angles (measured in degrees) were derived from sensors placed over T12, L3 and S2 using 3-Space^® ^Fastrak™ (Polhemus, Kaiser Aerospace, Vermont) and custom software written in LabVIEW V8 (National Instruments, Texas, USA). LLx (L3-S2), ULx (T12-L3), and total lumbar (TLx) angles (T12-S2) were calculated, as previously defined (see Figure 2) and shown to possess excellent inter-trial reliability in sitting [6]. Reliability and validity of the Fastrak™ system for measuring spinal range of motion has been demonstrated [32,33]. The Fastrak™ system is widely used in clinical research, however there are limitations of externally fixated measurement devices which have been discussed is detail elsewhere [34]. Extension in the sagittal plane was assigned a positive value, and flexion a negative value.

For usual sitting and standing the mean angle of three trials (averaged over 5 seconds of data collection) was calculated and used for subsequent analysis. For range of motion, the mean peak angle of three trials (averaged over 5 seconds subject held position) was calculated for each of; maximal slumped sitting, sway standing and maximal forward and backward bending in standing was subtracted from the usual sitting or standing angle. The mean peak sagittal angles were calculated for the functional tasks. As there was no sustained hold during these tasks (except for the squat), the customised analysis software determined the peak sagittal flexion (or least sagittal extension) angle reached between the manually tagged start and finish of the task. Range of motion from the reference position of usual standing to the peak angle in each functional task also calculated to compare relative motion between LLx and ULx regions during these tasks.

Inter-trial reliability (from three trials for each subject) for all LLx, ULx and TLx repeated measures in this study were excellent. For the LLx spine, the mean ICC_(3,1) _was 0.97 (range: 0.93 – 0.99) and mean SEM was 2.0° (range: 0.5° – 2.5°). For the ULx spine, the mean ICC_(3,1) _was 0.94 (range: 0.87 – 0.99) and mean SEM was 2.1° (range: 0.5° – 3.1°). For the TLx spine, the mean ICC_(3,1) _was 0.95 (range: 0.87 – 0.99) and mean SEM was 2.8° (range: 0.6° – 4.7°).

### Statistical Analysis

As this study was part of a larger prospective study, sample size calculations were not specific to this study. However, calculations using Intercooled Stata 9.2 for Windows (Statacorp LP, College Station: USA) indicated over 99% power to detect half of one standard deviation difference in range of motion between ULx and LLx angles within the 170 subjects (even when assuming a strong correlation of 0.9 between ULx and LLx angles). All other statistical analyses were performed using SPSS Student Version 13.0 (SPSS, Chicago: USA). A series of repeated measures ANCOVA for each posture or task, with the within-subject contrast being lumbar region, and the between-subject contrast being pain group, adjusting for BMI were used. For each task, the partial correlation between lumbar, regions adjusted for BMI, were calculated. The criteria for statistical significance was set at p < 0.05.

## Results

In usual sitting posture, the LLx spine was on average in an extended position, while the ULx spine was on average held in a slightly flexed (kyphotic) position. These mean LLx and ULx angles were significantly different (F = 28.23, p < 0.001). However, BMI was positively and significantly correlated with both LLx (r = 0.238, p = 0.002) and ULx (r = 0.203, p = 0.008) position. After adjusting for BMI, LLx and ULx angles were not significantly different (F = 0.46, p = 0.497). As shown in Table 2, the same pattern was seen with slump sitting, where ULx and LLx differences were no different after adjusting for BMI. Correlations between LLx and ULx angles are reported adjusted for BMI, however BMI had minimal effect on these correlations In usual and slump sitting, subjects' LLx angle showed no correlation with ULx angle.

**Table 2:** Comparisons between upper lumbar and lower lumbar spine static and peak angles across postures and tasks.

**Posture/Activity**	**ULx angle (°)**	**LLx angle (°)**	**p-value**	**p-value adjusted for BMI**	**ULx/LLx correlation (p-value)**
**Usual sitting**	4.1 ± 8.8	-1.3 ± 8.8	< 0.001	0.497	0.036 (0.638)
**Usual standing**	23.4 ± 11.2	15.5 ± 9.6	< 0.001	0.016	- 0.505 (< 0.001)
**Slump sitting**	1.6 ± 9.1	-8.6 ± 6.1	< 0.001	0.770	- 0.111 (0.151)
**Sway standing**	31.2 ± 13.6	17.4 ± 11.9	< 0.001	0.576	- 0.58 (< 0.001)
**Maximal flexion**	11.8 ± 6.8	15.4 ± 5.9	< 0.001	0.026	- 0.062 (0.426)
**Maximal extension**	44.1 ± 19.9	25.8 ± 16.0	< 0.001	0.183	- 0.509 (< 0.001)
**Pick up pen**	-8.1 ± 7.2	-12.5 ± 6.3	< 0.001	0.015	0.197 (0.012)
**Pick up box**	-5.3 ± 8.3	-8.5 ± 8.5	< 0.001	0.108	0.274 (< 0.001)
**Transfer pillow**	3.5 ± 8.5	-4.7 ± 8.3	< 0.001	0.013	- 0.017 (0.825)
**Transfer box**	8.4 ± 8.9	1.7 ± 8.4	< 0.001	0.031	- 0.147 (0.062)
**Squat**	-3.2 ± 9.0	-2.9 ± 9.6	0.866	0.968	0.346 (< 0.001)

Repeated measures ANCOVA and correlations adjusted for BMI.

LLx = Lower Lumbar, ULx = Upper Lumbar, BMI = Body Mass Index, Negative value = relative flexion (kyphosis) of lumbar spine.

In usual standing, both the LLx and ULx angles were on average in an extended (lordotic) position. BMI was not correlated with LLx position (r = -0.023, p = 0.767) but was positively and significantly correlated with ULx position (r = 0.194, p = 0.011). Even after adjusting for BMI, there was significantly more extension in the LLx spine than the ULx (see Table 2). The same pattern of more LLx extension was seen with sway standing and maximal extension in standing, but these differences were not significant after adjusting for BMI. In usual and sway standing and maximal extension, subjects' LLx angle showed a moderate inverse correlation with ULx angle.

The TLx sagittal range of motion (difference between maximal forward and backward bending angles) in standing was on average approximately 96° for all subjects, with a significantly greater proportion (58% v 42%) of this in the LLx spine compared with the ULx spine (F = 4.203, p = 0.042). BMI was positively correlated with LLx motion (r = 0.172, p = 0.025) but negatively correlated with ULx motion (r = -0.508, p < 0.001).

When changing postures from both sitting and standing positions, the LLx and ULx spine displayed different patterns of movement across all subjects. With usual sitting posture as the reference angles, when moving from usual sitting to slump sitting, the majority of movement occurred at the ULx spine, with significant differences between LLx and ULx movement [(F = 85.34, p < 0.001). However, BMI was negatively correlated with LLx motion (r = -0.313, p < 0.001) but not correlated with ULx motion (r = 0.056, p = 466) and after adjustment for BMI the differences between LLx and ULx movement were not significant at the critical alpha level [(F = 3.28 p = 0.072) see Table 3]. Conversely, with usual standing posture as the reference angles and adjusting for BMI, there was significantly more LLx movement compared to ULx movement when moving from; 1. Usual standing to maximal forward bending, 2. Usual standing to maximal backward bending, and 3. Usual standing to a sway standing posture. For the 2^nd ^and 3^rd ^task, BMI was positively correlated with LLx motion (r = 0.257, p = 0.001 and r = 0.327, p < 0.001 respectively) but negatively correlated with ULx motion (r = -0.343, p <0.001 and r = -0.477, p < 0.001).

**Table 3:** Comparisons between upper lumbar and lower lumbar spine range of motion across postures and tasks.

**Posture/Activity**	**LLx angle (°)**	**ULx angle (°)**	**p-value**	**p-value adjusted for BMI**	**ULx/LLx correlation (p-value)**
**Usual to slump sitting**	2.5 ± 4.0	7.3 ± 7.0	< 0.001	0.072	0.525 (< 0.001)
**Usual to sway standing**	8.2 ± 5.4	0.8 ± 5.4	< 0.001	< 0.001	- 0.469 (< 0.001)
**Usual stand to maximal flexion**	35.0 ± 10.0	30.8 ± 9.9	0.003	0.033	- 0.442 (< 0.001)
**Usual stand to maximal extension**	20.6 ± 13.3	8.9 ± 11.1	< 0.001	0.001	- 0.426 (< 0.001)
**Total standing ROM**	55.7 ± 18.6	39.8 ± 17.0	< 0.001	0.042	- 0.460 (< 0.001)
**Usual stand to pick up pen**	31.4 ± 9.9	28.0 ± 9.1	0.004	0.140	- 0.332 (< 0.001)
**Usual stand to pick up box**	28.7 ± 10.2	24.0 ± 10.1	< 0.001	0.069	- 0.142 (0.071)
**Usual stand to transfer pillow**	19.9 ± 8.2	20.3 ± 9.3	0.775	0.290	- 0.186 (0.018)
**Usual stand to transfer box**	15.0 ± 7.3	13.9 ± 8.2	0.170	0.160	- 0.041 (0.601)
**Usual stand to squat**	26.5 ± 10.2	18.4 ± 11.8	< 0.001	0.009	- 0.008 (0.918)

Repeated measure ANCOVA and correlations adjusted for BMI.

LLx = Lower Lumbar, ULx = Upper Lumbar, BMI = Body Mass Index, ROM = Range of Motion.

For the functional tasks, statistically significant differences were found between LLx and ULx peak angles for picking up a pen, picking up a box, transferring a pillow, and transferring a box, but not for squatting. However, after BMI adjustment, differences were only significant for picking up a pen and transferring a pillow and a box (Table 2). BMI was not correlated with these measures.

When comparing the differences in how far the LLx and ULx spine moved from the reference usual standing position to the peak angle position during functional tasks, picking up a pen, lifting a box from the floor, and squatting tasks all involved significantly more movement in the LLx spine. Only the difference in squatting remained significant after adjusting for BMI however (Table 3).

### Effect of LBP

TLx maximal backward bending range of motion was the only measure that was significantly different between pain groups (F = 5.18, p = 0.007). Significant LBP was associated with decreased movement compared to No Pain (-3.7°, 95%CI: -6.3° to -1.0°) or Mild Pain (-3.1°, 95%CI: -5.3° to -1.0°), and these estimates were unaffected by BMI. However, low back pain did not modify regional differences in any lumbar spine angle or range of motion before or after adjustment for BMI. Correlations between LLx and ULx were similar between pain groups across all tasks.

## Discussion

This study supports and extends previous literature that found global lumbar spine kinematics do not accurately reflect kinematics of the ULx or LLx spinal regions [6,19,35]. Rather the ULx and LLx spine display some functional independence and for the purposes of investigation of spinal posture, motion and loading, these regions should be considered separately.

### Sitting

The lack of correlation between LLx and ULx angles in usual sitting is consistent with a previous investigation of sitting posture [6] and supports the concept of regional differences. On average, the LLx spine in usual sitting was slightly extended, while the ULx spine was slightly flexed. When moving from a usual sitting to slump sitting position, the majority of motion occurred in the ULx spine, which also confirms the findings of Dankaerts et al using similar sitting protocol [6]. This movement from usual to slump sitting showed a moderate positive correlation, which is consistent with both lumbar regions moving towards their end of range flexion position.

These differences in LLx and ULx spine posture and motion in sitting were accounted for by subject's BMI, as BMI was positively correlated with LLx and ULx angles and this may be an indication that the body adapts its position in response to load. There is some evidence of BMI modifying posture and movement of the lumbar spine [18], and different movement strategies from sitting to standing have been reported between obese and normal individuals [36]. Other possible examples of the body adapting its position in response to load are the reduction in sitting and standing sagittal thoraco-lumbar motion with pregnancy [37] and self reported improvements spinal pain and posture following breast reduction surgery [38,39].

### Standing

In usual standing posture, there was more extension in the LLx than ULx segments. These angles showed a moderate inverse correlation, supporting their functional difference. Across all subjects, total sagittal range of motion in standing was similar to results reported in other studies [40,41] and the finding of a greater proportion of this motion occurring in the LLx spine is also consistent with previous findings [42,43].

Regional differences were also evident in lumbar movements from usual standing to positions of forward and backward bending as well as sway standing, with the majority of motion occurring at the LLx spine. Although previously clinically hypothesized [4,44], this study provides quantitative data that supports the idea that movement into the sway standing position is primarily a function of extension motion through the LLx segments, with very little motion occurring in the ULx spine. If adopted habitually, this sway standing position may result in increased load on passive spinal structures in the LLx spine due to inhibition of supporting spinal muscles [31], and may be a possible mechanism for LLx spine pain in some individuals.

Similar to sitting measures, BMI could account for some of the regional differences in static standing angles, particularly sway and maximal extension in standing. This finding is consistent with a recent study showing higher BMI was related to hyper-lordotic standing posture in adolescents [45]. BMI was moderately negatively correlated with ULx measures and positively correlated with LLx measures, particularly in a number of the range of motion measures. This suggests as BMI increases, ULx motion decreases and LLx motion increases, which supports and extends the findings of Gilleard and co-workers in a study comparing obese and normal individuals [18].

### Functional Tasks

Regional lumbar spine differences are supported by Gill et al's findings of LLx angle in healthy controls remaining consistent across different lifting techniques [19]. In their study, dynamic spinal position changes occurred at the ULx and thoracic spine. The current study adds to these findings, as there was a lack of correlation between LLx and ULx peak angles in the lifting tasks at bed height. Further, LLx and ULx range of motion from the reference position of usual standing to the peak angle in each functional task was either negatively correlated or showed no correlation. There were also significant differences between LLx and ULx peak sagittal angles across all tasks except squatting. Again BMI influenced these findings. Although the role of BMI in spinal posture and function requires further investigation, the results of this study clearly support that regional lumbar posture is influenced by BMI.

### The influence of LBP

There was a considerable prevalence of LBP reported in this relatively young sample of female undergraduate nursing students. Although not necessarily disabling, over 30% of the students had LBP that would be regarded as clinically significant. Given the supposed risk for LBP in nurses in relation to bending and lifting duties [46], this group of nursing students provided an interesting cohort for investigation of the influence of LBP on regional lumbar posture.

Whilst there were clear regional differences in both posture and motion observed in this study, there were no differences in these variables between subjects with and without LBP. This data suggests regional spinal angles do not differ in female nursing students with LBP when they are sub-grouped according to LBP severity. This finding conflicts with other gender controlled evidence that individuals with LBP stand with less LLx lordosis [47], or greater lower lumbar lordosis than healthy controls [48]. These conflicting results may be due to methodological differences, or alternatively may indicate that the manner by which LBP subjects are sub-grouped greatly influences whether postural differences are detected [4,6].

There is evidence for both a loss of segmental lordosis and excessive lower lumbar lordosis in different sub-groups of chronic LBP patients when classified on the basis of directional pain provocation [6,49]. Determining appropriate sub-classification of non-specific LBP populations appears to be a consensus in LBP research findings [50]. In the current study, sub-classification by LBP severity may have failed to adequately distinguish between LBP postural sub-groups, creating a wash-out effect [51]. Based on previous research [41,47,52], it is unclear whether the influence of gender on spinal posture or mobility also needs to be considered when interpreting these results.

Only total lumbar sagittal extension motion differed between LBP and control subjects, possibly suggesting spinal range of motion may not be important in LBP in this population. This may relate that subjects did not have high levels of current pain at the time of testing. Previous studies have reported reduced sagittal range of motion in LBP subjects compared with healthy controls [35,53,54]. However other studies suggest segmental hypermobility is present in LBP populations [55], or that both segmental hypermobility and segmental rigidity are evident in different sub-groups of LBP patients [56]. Clearly, variable definitions of LBP and different methods of measuring spinal angles (MRI, X-ray, external motion analysis systems) may account for some of these conflicting findings. Alternatively, other factors such as spinal motor control [4,57], habitual posturing of the spine [5,6], patterns of spinal loading [58], neurophysiological [59], psycho-social [60,61], and genetics [62] may be more important mediating factors of LBP experience than spinal range of motion, depending on the study population.

Interestingly, BMI did not influence the findings in relation to LBP in this study. This may be related to the lack of group differences in mean BMI scores and that the majority of subjects were within normal BMI range. In contrast, BMI has been associated with LBP in some studies [63,64], and evidence of BMI differences between standing postural alignment groups [45] may relate to compensatory patterns of loading due to body mass distribution. Given trends of increasing population obesity [65], the influence of BMI on LBP may become a greater issue in the future.

### Limitations

The results of this study of a moderate size cohort of young female nursing students cannot be generalised across other populations without further research. Particularly, the possibility of different findings between males and females across some of the measures warrants further investigation. The 3-dimensional motion analysis system is not a direct measure of spinal posture, however in a large clinical sample it is a widely accepted tool for the measurement of dynamic functional spinal angles [34]. It also has some clinical validity as a measure of spinal posture, as Dankaerts et al [6] were able to use 3-dimensional motion analysis measures to discriminate between both sub-groups of LBP as well as healthy controls.

Measurement of "usual" spinal posture in the laboratory setting is difficult. While efforts were made to blind the subjects as to when measurements of their sitting and standing posture were being recorded, this is an acknowledged weakness of the study. However, a recent study of lumbo-pelvic kinematics showed that after being asked to assume a "usual" sitting posture, subjects did not significantly alter this posture over five minutes of data collection [52], which adds some validity for this being a measure of "usual" sitting posture.

## Conclusion

This study supports the concept of separate regions of posture and movement within the lumbar spine. LLx posture is not directly related to ULx posture, and knowledge about movement in one region does not inform about movement in the other. Some regional differences in spinal angles are influenced by BMI, supporting that weight distribution has an influence over spinal posture and movement. Static posture angles, range of motion and dynamic spinal angles during functional tasks were not influenced by LBP. Regional lumbar posture and its relationship with recurrent or future LBP episodes is the subject of ongoing prospective research.

## List of Abbreviations

LBP: low back pain; LLx: lower lumbar; ULx: upper lumbar; TLx: total lumbar; ODI: Oswestry Disability Index; VAS: visual analogue scale

## Competing interests

The authors declare that they have no competing interests.

## Authors' contributions

TM and PBO conceived the study. TM recruited participants and carried out data collection and analysis. TM retains copyright on all contents. PBO, AB and LS assisted with study design and manuscript preparation. AS provided statistical support and assisted with manuscript preparation. All authors read and approved the final manuscript.

## Pre-publication history

The pre-publication history for this paper can be accessed here:


